# Evaluation of the Mechanical Properties of Highly Oriented Recycled Carbon Fiber Composites Using the Vacuum-Assisted Resin Transfer Molding, Wet-Layup, and Resin Transfer Molding Methods

**DOI:** 10.3390/polym17101293

**Published:** 2025-05-08

**Authors:** Mio Sato, Yuki Kataoka, Masumi Higashide, Yuichi Ishida, Sunao Sugimoto

**Affiliations:** 1 Japan Aerospace Exploration Agency, Tokyo 181-0015, Japan; higashide.masumi@jaxa.jp (M.H.);; 2 HOSEI University, Tokyo 184-8584, Japan

**Keywords:** recycled CFRP, mechanical property, molding method

## Abstract

Recycling carbon-fiber-reinforced plastics (CFRPs) is crucial for sustainable material utilization, particularly in aerospace applications, where large quantities of prepreg waste are generated. This study investigated the mechanical properties of highly oriented recycled CFRP (rCFRP) molded using vacuum-assisted resin transfer molding (VaRTM), wet-layup, and traditional RTM methods. Recycled carbon fibers (rCFs) obtained via solvolysis and pyrolysis were processed into nonwoven preforms to ensure fiber alignment through carding. The influence of molding methods, fiber recycling techniques, and fiber orientation on mechanical performance was examined through tensile tests, fiber volume fraction (Vf) analysis, and scanning electron microscopy observations. The results indicated that the solvolysis-recycled rCF exhibited superior interfacial adhesion with the resin, leading to a higher tensile strength and stiffness, particularly in the RTM process, where a high Vf was achieved. Wet-layup molding effectively reduced the void content owing to autoclave curing, maintaining stable properties even with pyrolyzed rCF. VaRTM, while enabling vacuum-assisted resin infusion, exhibited a higher void content, limiting improvements in mechanical performance. This study highlights that tailoring the molding method according to the desired performance, such as increasing stiffness potential by enhancing Vf in RTM or improving tensile strength by improving fiber–matrix adhesion in wet-layup molding, is critical for optimizing rCFRP properties, providing important insights into sustainable CFRP recycling and high-performance material design.

## 1. Introduction

Most carbon-fiber-reinforced plastics (CFRPs) used in aerospace applications are manufactured using prepregs, which are sheets of fiber impregnated with uncured resin. CFRP with enhanced properties in specific directions can be produced by laminating prepregs according to the designed fiber orientation. However, this process generates a significant number of prepreg offcuts when sheets are cut into various orientations and placed in molds. Additionally, prepregs have a limited shelf life, leading to the disposal of expired materials at manufacturing sites. Currently, much of the prepreg waste and scrap generated during CFRP production is disposed of in landfills [1]. However, as the capacity for landfill disposal is limited and such methods are unsustainable from an environmental perspective, the establishment of an efficient recycling system for both manufacturing waste and end-of-life CFRP is urgently required. To recycle aerospace CFRP and reapply recycled CFRP (rCFRP) in aircraft structures, it is essential to ensure that the mechanical properties of the rCFRP remain sufficient. Therefore, it is necessary to maintain as much of the length of recycled carbon fibers (rCFs) as possible [2]. Long rCFs can be processed into nonwoven fabrics, making them suitable base materials for rCFRP production, which serves as an effective recycling method [3].

CFRP recycling methods can be broadly classified into three categories: mechanical recycling [4,5,6], pyrolysis [7,8,9,10], and solvolysis [11,12,13]. Among these, pyrolysis and chemical decomposition can recover relatively long rCFs, so they were chosen as the focus of this study. Following the extraction of rCFs, they can be reused in CFRP. However, their mechanical properties depend not only on the type of CF and resin but also on molding conditions, including resin transfer molding (RTM), vacuum-assisted RTM (VaRTM), and autoclave curing. Various studies have investigated the fabrication and mechanical properties of rCFRP using various molding methods. Oliveux et al. formed composite plates with randomly distributed discontinuous rCFs via chemical decomposition using VaRTM [13]. They compared these with composite plates made from virgin CFs and demonstrated that if the surface conditions and fiber alignment of the recycled fibers were well maintained, the resulting properties could compete with those of virgin fibers. Kim et al. used chopped rCFs with lengths of 6, 12, 18, and 24 mm to fabricate composite plates via VaRTM [14], revealing a significant tradeoff between mechanical reinforcement and electrical sensing capabilities depending on the fiber length. Sales-Contini et al. fabricated rCFRP using pyrolyzed rCFs, first using hand layup followed by VaRTM, and evaluated its mechanical properties [15]. They found that sizing degradation during the pyrolysis process affected the results of tensile tests more than those of flexural tests. The degradation of sizing improved the resin–fiber interfacial adhesion, leading to enhanced strength under tensile loads.

Although various studies have investigated the effects of specific rCF extraction and molding methods on mechanical properties, few have systematically compared different fiber types and molding conditions [16]. Furthermore, the factors influencing the quality and mechanical properties of rCFs after reprocessing them into composite materials remain poorly understood. Therefore, this study investigated and compared the mechanical properties of rCFRP fabricated using rCFs obtained through pyrolysis and chemical decomposition. The rCFs were processed into nonwoven fabrics and molded using VaRTM, RTM, and wet-layup methods. This study characterizes how the fiber recovery method, molding conditions, and fiber alignment contribute to the mechanical properties of rCFRP, with the goal of enhancing mechanical performance.

## 2. Materials and Methods

### 2.1. Highly Oriented rCF Nonwoven Fabric

**Figure 1:**
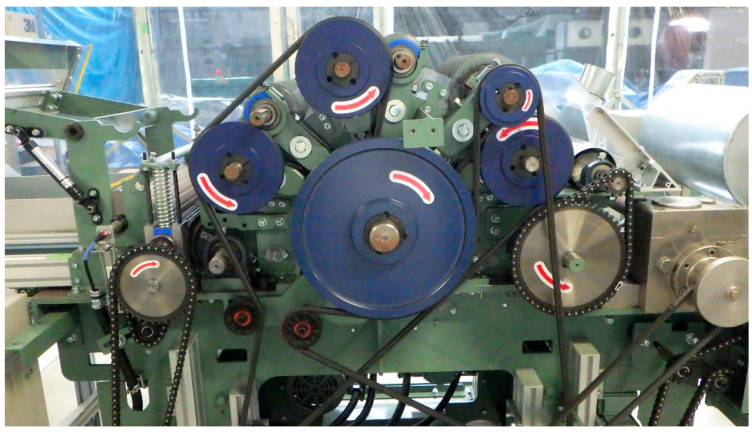
Carding machine (photograph taken by The Japan Wool Textile Co., Ltd.).

**Figure 2:**
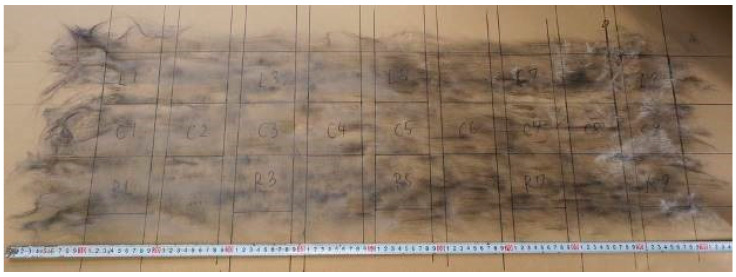
Preformed rCF nonwoven fabric (photograph taken by authors).

**Figure 3:**
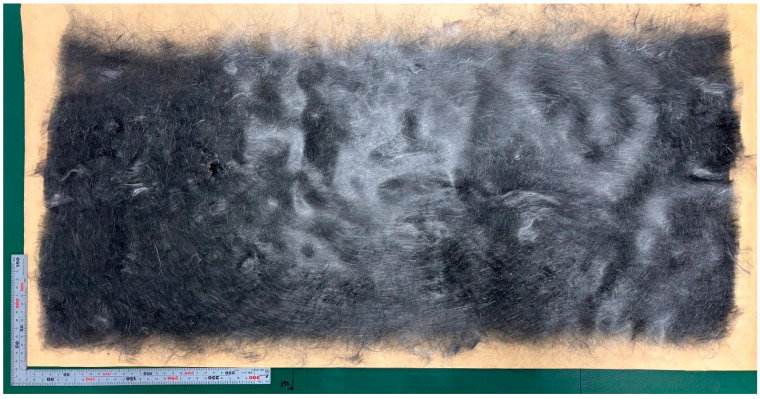
One ply of rCF nonwoven fabric (photograph taken by authors).

High-strength (high-quality) rCFRP is currently being developed in collaboration with JAXA (6-13-1 Osawa, Mitaka-shi, Tokyo, Japan) and The Japan Wool Textile Co., Ltd. (400 Sendo, Yoneda-chō, Kakogawa-shi, Hyogo, Japan). rCFs with an average fiber length of 80 mm obtained through either solvolysis or pyrolysis were carded using a carding machine, as shown in Figure 1. The fibers were processed to achieve an areal weight of 30 to 40 g/m^2^, forming a dry CF nonwoven preform, as shown in Figure 2. Several layers of these preforms were stacked to create a single ply of the nonwoven fabric, as shown in Figure 3. The resulting nonwoven fabric had a width of 350 mm and length of approximately 1 m. During the carding process, the fibers were highly oriented along the direction of carding, which was defined as the L-direction. Consequently, there was a noticeable difference in strength between the L-direction and transverse direction (T-direction).

### 2.2. VaRTM, Wet-Layup, and RTM Methods

For VaRTM and wet-layup molding, nonwoven fabrics were prepared using rCFs extracted through solvolysis and pyrolysis. After applying sizing to a one-ply rCF nonwoven fabric (approximately 350 mm square), the fibers were stacked in 10 plies while rotating each layer by 180° in the L-direction to account for the fiber alignment induced by the carding process. The matrix used was a thermosetting epoxy resin (Nagase ChemteX XNR 6809, cured at 120 °C according to the manufacturer catalog) with XN1233B as the curing agent for VaRTM and XNH 6809 for wet-layup, with a resin-to-hardener weight ratio of 100:90 for VaRTM and 100:95 for wet-layup. Resin impregnation was performed manually during the wet-layup process.

For VaRTM, two rCFRP plates were fabricated using solvolysis- and pyrolysis-treated rCF. Test specimens were prepared from each plate in both the L- and T-directions. For wet-layup molding, two rCFRP plates were prepared for each rCF treatment type and simultaneously cured in an autoclave under 0.5 MPa of pressure.

For RTM, a nonwoven fabric was prepared using solvolysis-treated rCF. After applying sizing to a one-ply rCF nonwoven fabric (150 mm wide and 200 mm long), 20 plies were stacked while rotating each layer by 180° in the L-direction. A binder was applied every five plies. The matrix used was a thermosetting epoxy resin (HUNTSMAN Araldite LY3585, cured at 90 °C according to the manufacturer catalog, with Aradur 3475 as the curing agent, with a resin-to-hardener weight ratio of 100:21), and the resin injection speed was set to 50 g/s.

### 2.3. Tensile Testing and Fiber Volume Fraction (Vf) Measurement

To verify the effectiveness of VaRTM and wet-layup molding, tensile testing specimens were prepared in the L- and T-directions. From the fabricated rCFRP plates, nine specimens were extracted in each direction with dimensions of 230 mm in length and 25 ± 0.1 mm in width. The average (Ave.) and standard deviation (S.D.) for sample thicknesses are listed in Table 1. Strain measurements were conducted using 5 mm strain gauges attached to both sides of the specimens.

**Figure 4:**
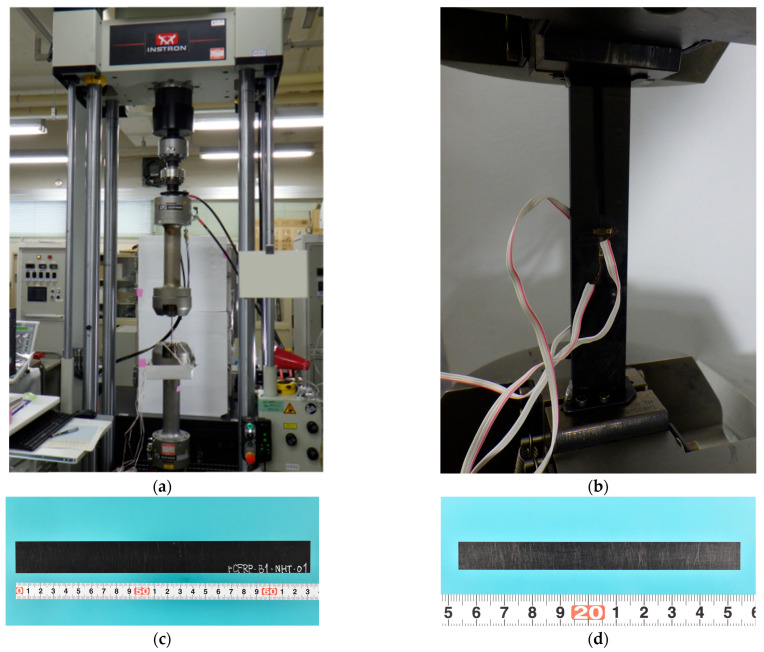
Tensile testing machine and samples. (**a**) Tensile test for VaRTM pyrolysis/L-direction, (**b**) magnified view of (**a**), (**c**) a sample of wet-layup pyrolysis/L-direction, and (**d**) a sample of RTM solvolysis/L-direction.

For the verification of RTM, tensile specimens were prepared from the rCFRP plates with dimensions of 100 ± 0.1 mm in length and 10 ± 0.1 mm in width. Ten specimens were prepared in the L-direction and six specimens in the T-direction. The thicknesses of the samples are listed in Table 1. The RTM process resulted in a particularly stable plate thickness. All tensile tests were conducted at a test speed of 1 mm/min by using INSTRON universal testing systems. The tensile testing machine and an example of a sample are shown in Figure 4.

A subset of the L-direction specimens was used to measure the fiber Vf using the combustion method in accordance with the JIS standard K 7075-1991 [17]. To account for variations in fiber density within the nonwoven fabric, 15 measurement points were selected from various locations across the samples. The calculations were performed using a water density of 0.998 g/cm³ and air density of 0.00118 g/cm³. The fiber Vf results were then used to compute the void volume fraction (Vv). The resin densities used for the calculations were 1.22 g/cm³ for XNR/H 6809 and 1.16 g/cm³ for Araldite LY3585 based on the values provided in the manufacturer catalogs.

## 3. Results and Discussion

### 3.1. Comparison of Vfs

**Figure 5:**
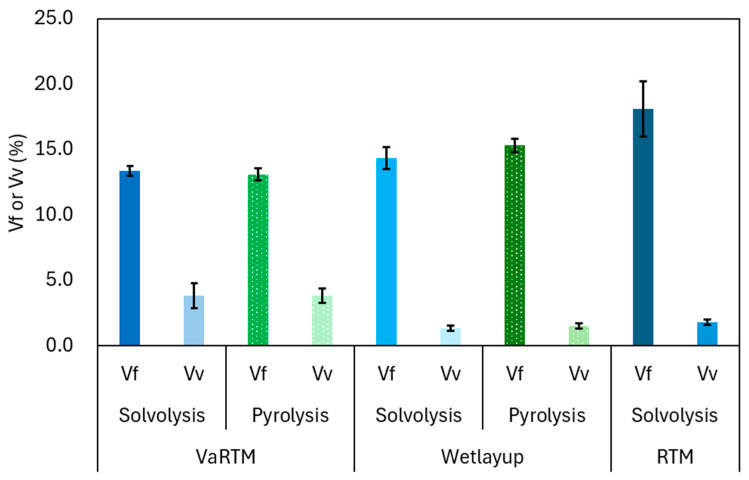
Vf and Vv results for different rCFRP specimens.

The results for the fiber Vf and Vv are presented in Figure 5. Because the RTM samples were fabricated with 20 plies, to eliminate the influence of the ply count, our analysis focused on VaRTM and wet-layup samples produced under comparable 10-ply conditions. In VaRTM, the vacuum-assisted resin infusion process ensures relatively uniform fiber content. However, owing to the inherent bulkiness of the nonwoven fabric, the applied external pressure is not effectively transmitted, leading to insufficient compaction and a relatively high Vv. In contrast, wet-layup molding incorporates an autoclave-based pressurized and heat-assisted curing process that effectively compresses and expels internal voids, thereby significantly reducing the Vv. Because RTM was performed using 20 plies, Vf increased due to the higher compaction pressure applied in this process. Additionally, the relatively large variation in Vf in the RTM samples suggests that fiber movement may have occurred during the resin injection process. In this phenomenon, the flow of resin causes localized fiber displacement and heterogeneity in fiber distribution. Such local variations in fiber volume fraction could diminish the expected anisotropic mechanical properties and may serve as initiation points for localized failure, thus adversely affecting the structural reliability of the final composite.

In general, nonwoven fabrics present challenges in terms of through-thickness resin impregnation. However, in this study, the Vv values of the RTM samples were relatively low (1.8%), indicating that effective resin impregnation was achieved. If VaRTM or wet-layup molding is performed with more than 20 plies, the bulkiness of the nonwoven fabric will likely lead to further insufficient compaction, making molding even more difficult. In contrast, RTM offers the advantages of high-pressure compaction and an automated resin impregnation process, allowing for fast and stable fabrication. Therefore, RTM is considered a promising method for increasing the Vf in CFRP using rCF nonwoven fabrics.

### 3.2. Comparison of Mechanical Properties

**Figure 6:**
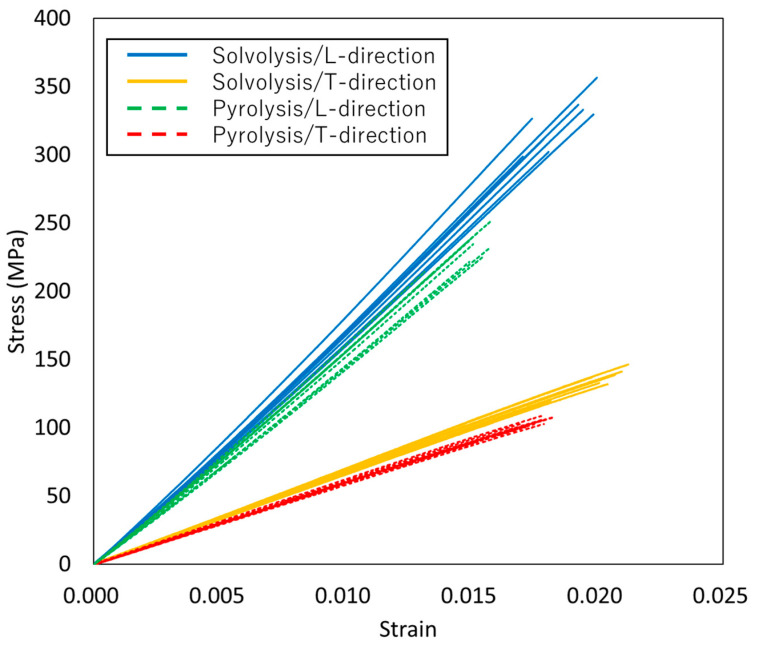
Stress–strain curves for VaRTM.

**Figure 7:**
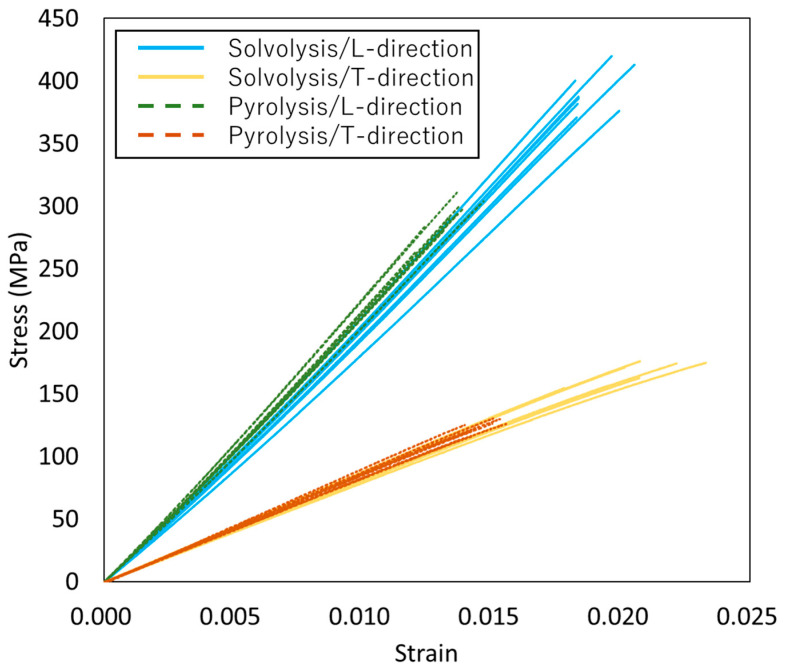
Stress–strain curves for wet-layup.

**Figure 8:**
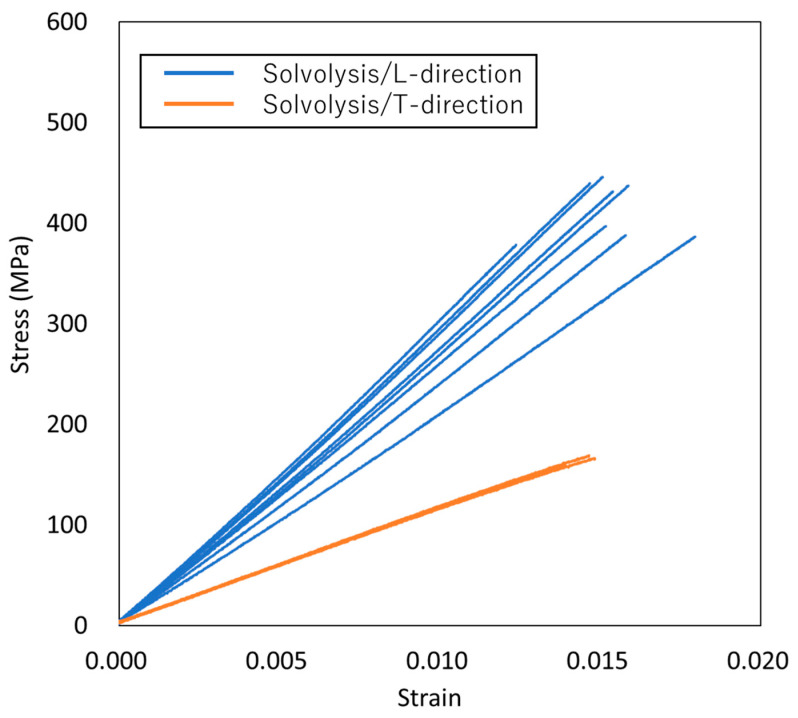
Stress–strain curves for RTM.

**Figure 9:**
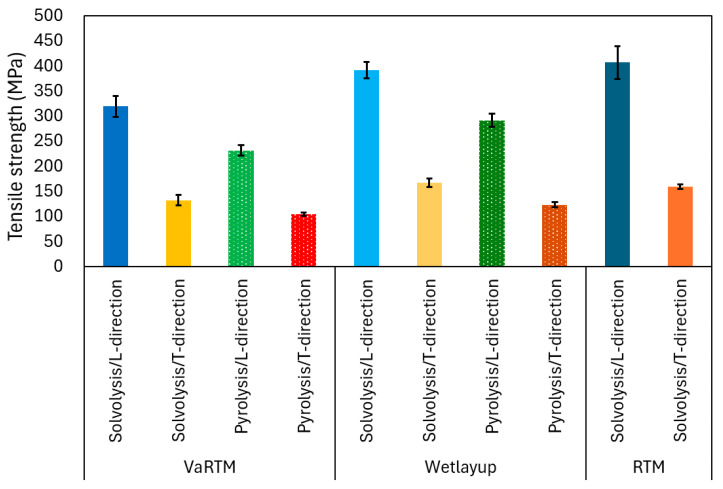
Influence of molding method on tensile strength.

**Figure 10:**
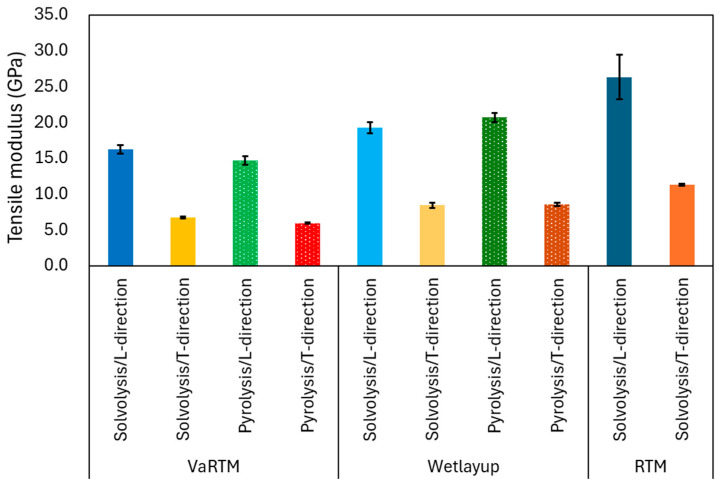
Influence of molding methods on elastic modulus.

**Figure 11:**
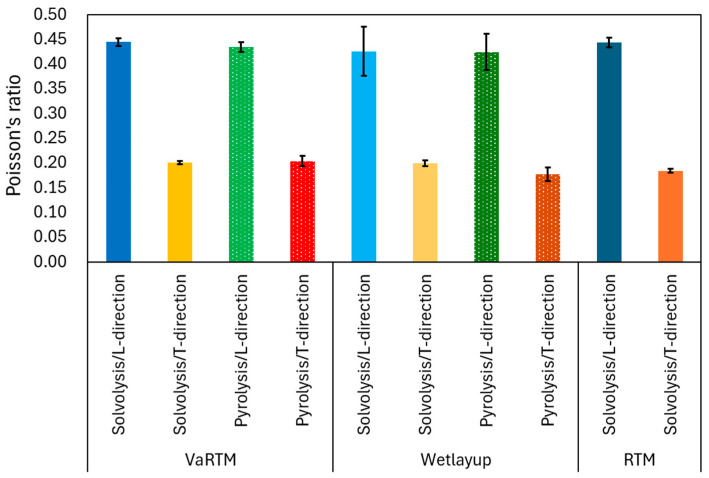
Influence of molding method on Poisson’s ratio.

The stress–strain curves for VaRTM, wet-layup, and RTM are presented in Figure 6, Figure 7 and Figure 8. Some test failures occurred during tensile testing: one case in the solvolysis/L-direction and two cases in the solvolysis/T-direction for wet-layup, and one case in the solvolysis/T-direction for VaRTM. These data were excluded from our analysis. As shown in Figure 6, Figure 7 and Figure 8, all samples exhibited linear relationships up to fracture, indicating brittle failure behavior. The fracture strain values are similar in the L- and T-directions, suggesting that the failure mode is independent of the fiber orientation, i.e., the final failure of the composite is governed primarily by the fiber strength rather than by fiber orientation effects. As shown in Figure 6 and Figure 7, the samples made from pyrolysis rCF tended to have lower fracture stress than those made from solvolysis rCF. These results align with previous findings indicating that pyrolysis rCF generally has lower fiber strength than solvolysis rCF [18]. This reduction in strength is likely attributable to fiber damage occurring during the pyrolysis process. However, in wet-layup molding, even samples using pyrolyzed rCF exhibited higher stiffness. This trend suggests that the pressurized curing process in the autoclave improved the fiber–resin interfacial adhesion and enhanced the overall stiffness. These results indicate that interface improvement contributes to the relatively high strength of the wet-layup-molded samples. The average values and standard deviations of the tensile strength, stiffness, and Poisson’s ratio obtained from the tests are summarized in Figure 9, Figure 10 and Figure 11. As shown in Figure 9 and Figure 10, in all cases, the elastic modulus and strength in the L-direction were approximately twice those in the T-direction, indicating that the mechanical properties in the L-direction are the best. This result can be attributed to the high fiber alignment in the carding direction introduced during the nonwoven fabric manufacturing process.

### 3.3. Influence of Molding Method on Tensile Strength

As shown in Figure 9, the RTM using 20 plies exhibited the highest L-direction tensile strength, particularly when using solvolysis rCF. This result can be attributed to the higher fiber Vf achieved under RTM conditions. A higher Vf increases the proportion of load-bearing fibers relative to the resin matrix, resulting in enhanced tensile strength by providing a more continuous and efficient stress transfer path. In wet-layup molding, although pyrolysis rCFs inherently have lower fiber strength due to thermal damage during the pyrolysis process, the application of pressure and heat curing in an autoclave significantly reduces the void content and improves fiber–matrix interfacial adhesion. The improved interface enables more effective load transfer from the matrix to the fibers, thereby maintaining relatively stable tensile strength. VaRTM was expected to reduce the void content through vacuum-assisted resin infusion, but it yielded a higher void fraction owing to the bulkiness of the nonwoven fabric. As a result, the fiber–resin interface continuity was compromised, reducing the stress transfer efficiency and limiting the L-direction strength improvement compared with wet-layup and RTM.

### 3.4. Influence of Molding Method on Elastic Modulus

As shown in Figure 10, the RTM achieved the highest L-direction elastic modulus, which can be attributed to its high Vf. Wet-layup molding with autoclave-assisted curing effectively minimized the void content and improved the fiber–resin interfacial adhesion, maintaining a high L-direction modulus. In contrast, VaRTM, which has a relatively high void fraction, disrupts the continuity of the load-bearing fiber network, thereby reducing the effective elastic modulus. In the T-direction, the influence of fiber orientation was minimal and the mechanical response was largely matrix-dependent, resulting in generally lower elastic modulus values. The differences between the fiber treatment methods and molding techniques were less pronounced in the T-direction than in the L-direction.

### 3.5. Influence of Molding Method on Poisson’s Ratio

As shown in Figure 11, the Poisson’s ratios in the L-direction ranged from 0.42 to 0.44, whereas in the T-direction, they ranged from 0.18 to 0.20 for all molding methods. Poisson’s ratio is sensitive to the fiber–matrix interfacial properties, fiber alignment, and microscopic structural heterogeneities. In the L-direction, the high stiffness of the rCFs plays a dominant role in axial loading, and the lateral strain behavior is significantly influenced by the fiber alignment, resin impregnation quality, and interfacial properties. A well-adhered fiber–matrix interface tends to suppress lateral contraction under axial loading, resulting in a relatively smaller Poisson’s ratio. Conversely, weak interfacial bonding or non-uniform fiber distribution allows the matrix deformation, leading to increased lateral strain and thus a higher Poisson’s ratio. A notable observation is the higher variation in Poisson’s ratio in wet-layup molding. Because the resin impregnation in wet-layup molding was performed manually during stacking, inhomogeneities in the resin distribution and fiber alignment within the layers may have contributed to the localized variations in the lateral strain behavior. These microstructural inconsistencies may have led to increased statistical variation in the Poisson’s ratio. The numerical values corresponding to Figure 5, Figure 9, Figure 10 and Figure 11 are listed in Table 2.

### 3.6. Scanning Electron Microscopy (SEM) Observation

**Figure 12:**
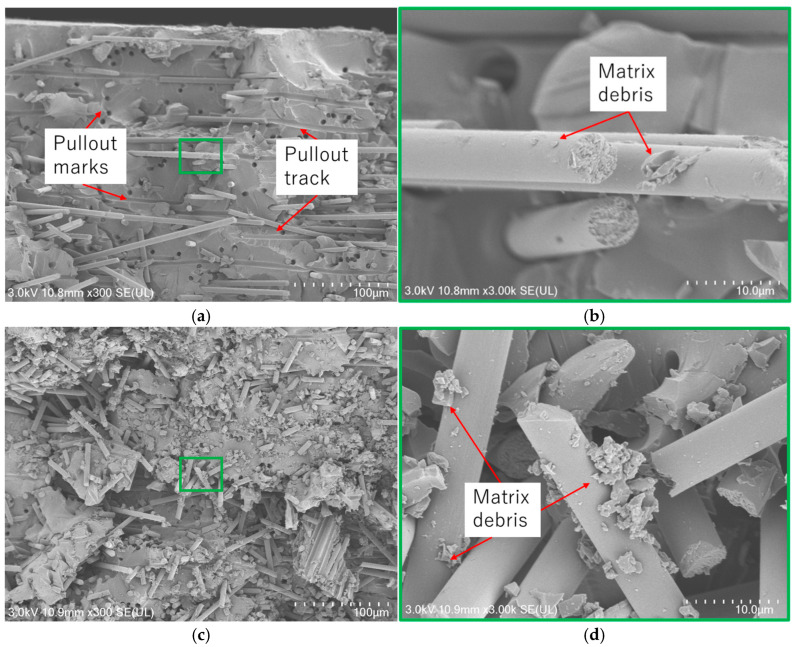
SEM observation of VaRTM solvolysis/L-direction and wet-layup solvolysis/L-direction samples. (**a**) VaRTM solvolysis/L-direction, (**b**) magnified view of (**a**), (**c**) wet-layup solvolysis/L-direction, and (**d**) magnified view of (**c**).

**Figure 13:**
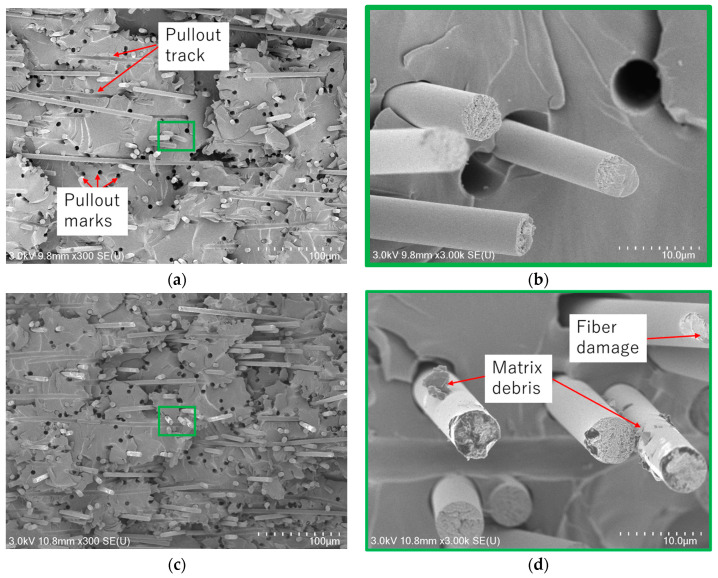
SEM observation of VaRTM pyrolysis/L-direction and wet-layup pyrolysis/L-direction samples. (**a**) VaRTM pyrolysis/L-direction, (**b**) magnified view of (**a**), (**c**) wet-layup pyrolysis/L-direction, and (**d**) magnified view of (**c**).

In the previous subsections, the mechanical properties of CFRP samples fabricated using VaRTM, wet-layup, and RTM were evaluated using rCFs obtained through pyrolysis and solvolysis. The results revealed that the fiber–matrix interfacial properties, fiber quality, and surface conditions of the rCF significantly influenced the mechanical properties of the rCFRP. To validate these findings, fracture surface observations were conducted using an FE-SEM Regulus8220 device. Figure 12 presents the L-direction tensile fracture surfaces of VaRTM and wet-layup samples fabricated using solvolysis rCF. The amount of matrix debris adhering to the fiber surfaces after fracture provides insight into the quality of the fiber–matrix interfacial bonding. A strong fiber–matrix interface typically results in cohesive failure within the resin itself during tensile loading, leaving resin debris attached to the fiber surfaces. Conversely, weak interfacial bonding promotes interfacial failure, leading to fiber pullout and the appearance of smooth fiber surfaces and pullout marks after fracture. In the case of the VaRTM samples, even though resin infusion was performed under relatively low pressure via vacuum assistance, good interfacial adhesion was achieved, and matrix debris remained on the fiber surface as a result of cohesive failure (Figure 12b). In wet-layup samples using solvolysis rCF, a greater accumulation of resin fragments around the fibers was observed, suggesting extremely strong interfacial bonding between the fiber and resin. Figure 13 presents the L-direction tensile fracture surfaces of VaRTM and wet-layup samples fabricated using pyrolysis rCF. In the VaRTM samples, the interfacial adhesion was found to be insufficient and fiber pullout marks, which indicate interfacial failure, were observed as the dominant fracture mechanism (Figure 13a). Additionally, high-magnification images revealed that the fiber surface appeared relatively smooth, further supporting the presence of weak interfacial adhesion. In contrast, in wet-layup samples using pyrolysis rCF, some resin fragments remained on the fiber surfaces, suggesting relatively improved interfacial bonding. However, it was also observed that the rCF surfaces exhibited thermal damage, which was likely caused by the pyrolysis process (Figure 13d). These SEM observations strongly support the findings of our mechanical property evaluation, confirming that the fiber surface condition and interfacial properties play a crucial role in determining both the failure mode and overall mechanical properties of CFRP.

## 4. Conclusions

In collaboration with JAXA and The Japan Wool Textile Co., Ltd., this study focused on the development of high-strength (high-quality) rCFRP. Using rCF nonwoven fabric, rCFRP plates were fabricated using wet-layup, VaRTM, and RTM methods, and the effects of the molding method, fiber recovery technique, and fiber orientation on mechanical properties were evaluated. The key findings of this study can be summarized as follows.

- Effect of CF Recovery Method:
  - rCFs recovered via solvolysis exhibited good surface conditions and improved fiber–matrix interfacial properties, leading to enhanced L-direction tensile strength and stiffness.
  - rCFs recovered via pyrolysis exhibited surface damage, which led to the deterioration of interfacial properties, resulting in lower strength and fracture strain, particularly in the L-direction.
- Effect of Molding Method:
  - RTM facilitated the highest Vf, particularly when using solvolysis rCF, leading to a significant improvement in tensile strength and elastic modulus in the L-direction.
  - Wet-layup molding incorporating autoclave curing effectively reduced the Vv and maintained relatively stable properties, even when pyrolysis rCF was used.
  - VaRTM, although expected to reduce voids through vacuum-assisted resin infusion, exhibited a higher residual void content owing to the bulkiness of the nonwoven fabric, which limited the L-direction property improvement.
- Effect of Fiber Orientation on Anisotropy:
  - Owing to the fiber alignment introduced by carding, the mechanical properties in the L-direction were approximately twice as high as those in the T-direction, confirming significant anisotropy.
  - Regardless of the fiber orientation, all samples exhibited brittle failure and there was no significant directional dependence on the fracture strain.
- Verification of Interfacial Properties through SEM Observations:
  - In the samples fabricated with solvolysis rCF, a greater amount of residual resin was observed on the fiber surfaces, confirming good interfacial adhesion.
  - In the VaRTM samples using pyrolysis rCF, interfacial failure was dominant, resulting in clean fiber surfaces at the fracture plane. In contrast, wet-layup molding yielded some interfacial improvement, as indicated by the partial resin adhesion on the fiber surfaces. These SEM findings are consistent with the mechanical property evaluations.

These results demonstrate that the quality of rCFs and the molding conditions significantly influence the mechanical properties and failure behavior of rCFRP. The insights gained from this study provide valuable guidelines for optimizing the design and manufacturing processes of highly oriented rCFRP.

## Figures and Tables

**Table 1:** Sample thicknesses.

Molding Method	rCF and Direction	Thickness (mm)	
		Ave.	S.D.
VaRTM	Solvolysis/L-direction	2.46	0.078
	Solvolysis/T-direction	2.60	0.030
	Pyrolysis/L-direction	2.73	0.167
	Pyrolysis/T-direction	3.00	0.049
Wet-layup	Solvolysis/L-direction	2.01	0.057
	Solvolysis/T-direction	2.08	0.024
	Pyrolysis/L-direction	1.78	0.034
	Pyrolysis/T-direction	1.79	0.038
RTM	Solvolysis/L-direction	2.07	0.010
	Solvolysis/T-direction	2.06	0.004

**Table 2:** Summary of testing results.

		Tensile Strength (MPa)		Tensile Modulus (GPa)		Poisson’s Ratio		Vf (%)		Vv (%)	
		Ave.	S.D.	Ave.	S.D.	Ave.	S.D.	Ave.	S.D.	Ave.	S.D.
VaRTM	Solvolysis/L-direction	320	21.1	16.2	0.6	0.44	0.008	13.3	0.4	3.8	1.0
	Solvolysis/T-direction	133	10.3	6.7	0.2	0.20	0.003	-	-	-	-
	Pyrolysis/L-direction	232	10.8	14.7	0.6	0.43	0.009	13.1	0.4	3.8	0.6
	Pyrolysis/ T-direction	105	2.7	6.0	0.1	0.20	0.011	-	-	-	-
Wet-layup	Solvolysis/ L-direction	392	16.4	19.3	0.8	0.43	0.050	14.4	0.8	1.4	0.2
	Solvolysis/ T-direction	167	8.4	8.4	0.4	0.20	0.005	-	-	-	-
	Pyrolysis/ L-direction	292	13.1	20.7	0.6	0.42	0.037	15.3	0.5	1.5	0.2
	Pyrolysis/ T-direction	124	5.5	8.6	0.2	0.18	0.014	-	-	-	-
RTM	Solvolysis/ L-direction	407	32.5	26.3	3.1	0.44	0.009	18.1	2.1	1.8	0.2
	Solvolysis/ T-direction	160	4.2	11.3	0.1	0.18	0.004	-	-	-	-

## Data Availability

The data are not publicly available to prevent indiscreet replications.

## Abbreviations

The following abbreviations are used in this manuscript:

CFRP	Carbon-fiber-reinforced plastic
rCFRP	Recycled CFRP
VaRTM	Vacuum-assisted resin transfer molding
CF	Carbon fiber
rCF	Recycled carbon fiber
Vf	Fiber volume fraction
Vv	Void volume fraction
SEM	Scanning electron microscopy
